# Estimation of crossbreeding and genetic parameters for reproductive traits of Boer x Central Highland goats in Ethiopia

**DOI:** 10.1371/journal.pone.0291996

**Published:** 2023-09-27

**Authors:** Zeleke Tesema, Belay Deribe, Mekonnen Tilahun, Alemu Kefale, Getachew Worku Alebachew, Kefyalew Alemayehu, Tesfaye Getachew, Damitie Kebede, Mengistie Taye, Solomon Gizaw

**Affiliations:** 1 Debre Birhan Agricultural Research Center, Debre Birhan, Ethiopia; 2 Sirinka Agricultural Research Center, Woldia, Ethiopia; 3 Andasa Livestock Research Center, Bahir Dar, Ethiopia; 4 College of Agriculture and Environmental Sciences, Bahir Dar University, Bahir Dar, Ethiopia; 5 International Center for Agricultural Research in the Dry Areas (ICARDA), Addis Ababa, Ethiopia; 6 International Livestock Research Institute (ILRI), Addis Ababa, Ethiopia; Colorado State University, UNITED STATES

## Abstract

Additive genetic and non-additive parameters for reproductive traits of Boer x Central Highland goats were estimated. Pedigree and performance records comprised of Central Highland and their crosses with Boer goats were collected from 2009 to 2018 in the Sirinka Agricultural Research Center sheep and goat breeding station. Least-squares means for genotypes were obtained using a general linear model procedure in SAS. To estimate crossbreeding parameters, breed additive, heterotic, and recombination effects were fitted as fixed covariates instead of genotypes. Variance, heritability, and repeatability estimates were estimated through the AI-REML algorithm using WOMBAT software. Genotype did not significantly (P>0.05) influence most of the reproductive traits studied except for gestation length. The additive effect for litter size at birth (LSB), total litter birth weight (LBW), total litter weaning weight (LWW), litter size at weaning (LSW), and gestation length (GL) was estimated to be -0.004 kid, 0.08 kg, -3.18 kg, -0.54 kid, and 3.69 days, respectively. The contribution of heterosis to LSB, LWW, and GL of crossbred goats was negative, while the estimates for LBW and LSW were positive. However, Boer goats’ heterosis effect and direct additive contribution to reproductive traits were insignificant (P>0.05) except for LSW. The recombination effect was negligible and not significant (P>0.05) for all traits examined. The direct heritability estimate for LSB, LWB, LWW, LSW, and GL were 0.050, 0.098, 0.086, 0.018, and 0.00, respectively. The repeatability estimates for LSB, LWB, LWW, LSW, and GL were 0.149, 0.116, 0.099, 0.086, and 0.061, respectively. The result indicates that improvement in reproductive traits would not be expected by crossing Boer with Central Highland goats. In addition, heritability estimates indicate that the improvement of reproductive traits through selection will be small, and the repeatability estimates indicate that multiple records have to be used to make a decision of culling or selection.

## Introduction

Genetic improvement of indigenous goats through crossbreeding is a major way to enhance productivity quickly, as it exploits both additive and non-additive effects [1, 2]. Because of this, milk-type exotic breeds such as Anglo-Nubian, Saanen, Toggenburg, and meat-type Boer goats have been introduced to Ethiopia with the aid of different non-governmental and governmental institutions since 1975 [3]. Boer goat is known for their better growth rate and meat. On the other hand, indigenous goats are resistant to disease, heat, and drought tolerance. Thus, the Boer goat has crossed with Central Highland, Abergelle, and Woyito-Guji goat breeds of Ethiopia to combine productivity and adaptability through crossbreeding. The resulted in Boer crossbred goats being disseminated to smallholder farmers for crossing purposes.

Genetic evaluation of the performances of Boer crossbred goats is quite important for further expansion of that genotype or changing to the appropriate crossbreeding scheme or breeding system for the future [4–6]. Differences among breed effects relative to magnitudes of heterosis and recombination effects are the major determinants of the efficiency of various crossbreeding systems [7]. Thus, for the planning of an effective crossbreeding program, information on the relative performances of breeds and their crosses, particularly under various environmental conditions, is also required [8]. Indeed, estimates of genetic parameters for the reproductive traits of different goat breeds have been reported by several scholars [9–14]. However, the estimates are affected by breed, genetic composition in the population [9], management, and physical environment. Besides, regarding the Boer goat, the previous studies in Ethiopia are limited to performance evaluation, identifying non-genetic factors [5, 6, 15, 16], and estimating genetic parameter estimates for growth traits [4]. A review of the literature by the authors showed that there needs to more estimates of genetic and crossbreeding parameters such as additive, heterosis, and recombination effects for the reproductive traits of Boer x Central Highland goats. Therefore, this study aimed to estimate the crossbreeding and genetic parameters for the reproductive traits of Boer x Central Highland goats.

## Materials and methods

### Ethics statement

Prior to the study, data collection formats and procedures were reviewed and approved by the Researchers of Amhara Regional Agricultural Research Institute, Ethiopia (number Ls/Ru-3/Sr-2015/19) in the annual review forum. Besides, this study was based on data collected from live goats managed at Sirinka Agricultural Research Station without any invasive procedure through close monitoring of researchers. Anesthesia, euthanasia, or animal sacrifice was not part of the study.

### Animal management

The study was conducted at Sirinka Agricultural Research Center, located at an altitude of 1850 m.a.s.l and 11°45’ 00" N and 39°36’ 36" E. The mean annual rainfall amount is, on average, about 950 mm. The area has a mean daily temperature range of 13.7 to 26.4°C, making it a moderately warm climate zone. A semi-intensive management system, defined by a moderate amount of production inputs, was used to handle the animals. They were allowed to graze or browse on the natural pasture during the daytime for approximately six hours and in addition, supplemented with 0.10 to 0.40 kg concentrate mixture (consisting of wheat bran, *Noug* seed cake, and salt) day^-1^ depending on their age, physiology, and sex. Animals were housed according to their sex, physiological status, sex, and health status. They received water *ad libitum* and were vaccinated against common diseases in the area, treated, de-wormed, and sprayed regularly. Each kid was given a unique identifying number, and the birth weight was determined within 24 hours of birth. Kids were kept indoors for 3–5 days with their dams, and after a maximum of five days, dams were kept outside, and kids were allowed to suckle three times a day until weaning age (90 days).

A single sire was assigned for 20–30 does for a service period covering two estrous cycles. Indigenous Central Highland does were mated with pure Boer bucks to produce F1 crossbreds with 50% Boer level. F2 crossbreds were produced through *inter se mating* of F1 males and females. Female crossbred progenies with 50% Boer level were crossed with pure Boer bucks to upgrade to a high Boer level (75%). To create crossbreds with a 25% Boer level, pure female Central Highland goats and crossbred bucks with a 50% Boer level were crossed [6]. Tesema et al. [4] and Tesema et al. [5] have reported a detailed description of flock management.

### Data and studied traits

Pedigree information and performance records related to reproductive traits of 567 does comprise Central Highland (CH) and their crosses with Boer (B) goats were collected from 2009 to 2018 in the Sirinka Agricultural Research Center shoat breeding station. The crossbred does include four genetic groups: 50% F1 B x CH, 50% F2 B x CH, 25% BCH x CH, and 75% B x BCH back cross one. The number of goats in each genotype group is shown in Table 2. The dataset includes the animals, sire, dam, mating date, kidding date, kid sex, type of birth, parity of dam when kidding, and records of live weight of kids at different ages. The studied traits include litter size at birth (LSB), total litter birth weight (LBW), litter size at weaning (LSW), total litter weight at weaning (LWW), and gestation length (GL). The weaning age of kids in this study was 90 days. LSB is the number of kids born per doe, LBW is the total weight of kids born per doe, LSW is the total number of kids weaned per doe, LWW is the total weight of kids weaned per doe, and GL is the number of days between mating and kidding.

### Statistical analysis

Least-squares means for the effect of genotypes were obtained using a general linear model procedure in SAS [17] with fixed effects of genotype, year of kidding, season of kidding, birth type, sex of kids, and dam parity. The statistical model for least square mean estimation is presented as follows:

$$Y_{ijklmno}=\mu +A_{i}+B_{j}+D_{k}+T_{l}+G_{m}+F_{n}+e_{ijklmno}$$ (1)

Where Y_ijklmno_ is the dependent variables, μ is the overall mean, A_i_ is the effect of i^th^ parity of doe (five classes: 1, 2, 3, 4, and ≥5), B_j_ is the effect of j^th^ genotype (five classes: CH x CH, F1 B x CH, F2B x CH, B x BCH, and BCH x CH), D_k_ is the effect of k^th^ year of kidding (nine classes: 2009–2018), T_l_ is the effect of l^th^ season of kidding (three classes: dry, main rain, and short rain), G_m_ is the effect of m^th^ sex of kid (two classes: male and female), F_n_ is fixed effect of n^th^ birth type (two classes: single and multiple), and e_ijklmno_ is random error term associated with each observation.

A multiple regression approach developed by Robison et al. [18] was used to estimate the crossbreeding parameters. The coefficient of expected breed additive (g_i_), heterozygosity (h_ij_), and recombination (r_ij_) effects were fitted as covariates to compute breed additive, heterosis, and recombination loss. In addition to these covariates, the fixed effects in model 1, except genotype, were fitted. The breed additive effect for Boer was estimated as deviations from the Central Highland goat breed. The expected coefficients used for crossbreeding parameter estimation for doe productive traits (Table 1) were derived according to the procedure of Dickerson [19] and Wolf et al. [20] as follows:

$$g_{i}=1/2({ɑ}^{s}{}_{i}+{ɑ}^{d}{}_{i})$$ (2)

$$h_{ij}={ɑ}^{s}{}_{i}{ɑ}^{d}{}_{j}+{ɑ}^{s}{}_{j}{ɑ}^{d}{}_{i}$$ (3)

$$r_{ij}=4g_{i}g_{j}‐h_{ij}$$ (4)

**Table 1:** Genetic coefficient used for crossbreeding parameter estimation for doe productive traits.

Genetic group	Generation and blood level	g	h	r
CH x CH	Local	0	0	0
B x CH	F1 (50%)	0.50	1.00	0
B x BCH	BC1 (75%)	0.75	0.50	0.25
BCH x CH	BC1 (25%)	0.25	0.50	0.25
BCH x BCH	F2 (50%)	0.50	0.50	0.50

B, Boer goat; CH, Central Highland goat; the sire’s breed is mentioned first

F1, first filial generation, F2, second filial generation; BC1, back cross one

g, breed additive; h, heterosis; r, recombination

Where g_i_ is breed additive, h_ij_ is heterozygosity, r_ij_ is recombination, ɑ^s^_i,_ and ɑ^d^_i_ denote the gene proportion of breed ‘i’ in the sire and dam of the animal, respectively. In addition, g_i_ is the breed additive effect, h_ij_ is heterosis, and r_ij_ is the recombination loss.

Without actually breeding the animals, it is crucial to estimate crossbreeding characteristics to predict the performance of untested genotypes and thus enable to choose breeding systems [19, 21]. Therefore, a prediction was made for each genotype examined for investigated traits according to Lynch and Walsh [22] and Demeke et al. [21]:

$$\bar{y}=\beta a$$ (5)

Where ***ȳ*** is the predicted mean for each genotype, ***β*** is the matrix of expected genetic contribution (breed additive, heterosis, and recombination loss), and ***a*** is a vector of estimated crossbreeding parameters, including the overall mean.

Variance components, heritability, and repeatability estimates were estimated through the AI-REML algorithm using WOMBAT software [23]. Since the number of does with records for each genotype was inadequate, genetic parameters could not be estimated separately. As a result, a dataset from a crossbred population born from 13 sires and 49 dams was used for genetic parameter estimation. The average number of offspring is 22 does/sire and 6 does/dam for LBW and LSB. Likewise, for LSW and LWW, the number of offspring per sire and dam was 21 and 6, respectively. Heritability (h^2^) = σ^2^_*a*_ / σ^2^_p_ and repeatability (r) = σ^2^_*a*_ + σ^2^_e_ / σ^2^_p_, where σ^2^_*a*_ is the direct additive genetic variance, σ^2^_e_ is error variance and σ^2^_p_ is phenotypic variance. The animal model used in the mixed model analyses of reproductive performance traits was the following:

$$Y=X\beta +Z_{a}a+Z_{c}c+e$$ (6)

Where **y** is the vector of observations for the dependent variable or reproductive traits (LSB, LBW, LSW, LWW, and GL); **X** is the incidence matrix of fixed effects of reproductive traits (factors mentioned above) and is the corresponding vector of fixed effects; ***Z***_***a***_ is the incidence matrix of the direct additive genetic effects; ***a*** is the vector of direct additive genetic effects associated with the ***Z***_***a***_ incidence matrix; ***Z***_***c***_ is the incidence matrix of the permanent effects of the dams; **c** is the vector of permanent environmental effects of dams associated with the **Z**_**c**_ incidence matrix; **e** is the vector of residual random effects associated with the observations. The direct additive effects, permanent environmental effects and residual effects were assumed to be uncorrelated and have expected means of zeros and variances σ^2^a, σ^2^c and σ^2^e, respectively, where var(a) = Aσ^2^a, var(c) = Iσ^2^c and var(e) = Iσ^2^e.

As a limitation, the sample size and data structure may influence the interpretation of the findings from this study to some extent. Indeed, the data was collected by researchers under on-station management of animals, which may reduce its impact on genetic and crossbreeding parameter estimates. Thus, this should be considered when using the results for heritability and repeatability.

## Result and discussion

### Genotype effect

The least-square means of reproductive traits for different genotypes are presented in Table 2. The overall mean for LSB, LBW, LWW, LSW, and GL were 1.54±0.02 kids, 3.90±0.05 kg, 14.0±0.35 kg, 1.37±0.02 kids, and 148.1±0.31 days, respectively. Tesema et al. [5] have previously reported the least-squares means for various fixed effects (including year, season, birth type, and sex) and their effect on doe-productive traits. Year of kidding, birth type, and dam parity significantly influenced most of the traits considered in this study. However, genotype had no significant influence on most of the reproductive traits investigated except for GL. CH x CH dams had an extended gestation length than BCH x CH dams. In terms of LSB, LWB, LWW, and LSW, indigenous goats (CH x CH) and crossbred does with 25% Boer level (BCH x CH) showed a tendency to perform better than other genotypes, although the difference was not significant (P>0.05). In line with this result, the absence of a significant difference in reproductive traits among indigenous and Boer crossbred dams was noted in several studies [24–26]. According to this result, the indigenous Central Highland dams tended to perform similarly in all traits to the crossbred dams, or crossing Boer goats with Central Highland goats were not improved the productive traits of the crossbred does significantly.

**Table 2:** Least-squares mean values and their standard errors for doe productive traits.

Genetic group	N	LSB (kid)	LBW (kg)	N	LWW (kg)	LSW (kid)	N	GL (day)
Overall mean	567	1.54±0.02	3.90±0.05	478	14.0±0.35	1.37±0.02	303	148.1±0.31
Significance		ns	ns		ns	ns		*
CH x CH	275	1.57±0.03	4.00±0.08	243	14.3±0.57	1.40±0.03	99	149.2±0.39^a^
B x CH	214	1.49±0.03	3.83±0.07	174	13.6±0.44	1.35±0.03	147	148.1±0.55^ab^
B x BCH	27	1.52±0.11	3.55±0.25	20	12.8±1.22	1.20±0.09	19	147.1±1.09^ab^
BCH x CH	19	1.73±0.10	4.08±0.31	17	15.4±2.58	1.47±0.12	12	145.2±0.53^b^
BCH x BCH	32	1.59±0.09	3.70±0.24	24	13.1±1.57	1.33±0.09	26	146.4±0.74^ab^

N, number of observation; ns, non-significant; LBW, litter birth weight; LSB, litter size at birth; LWW, litter weaning weight; LSW, litter size at weaning; GL, gestation length

B, Boer goat; CH, Central Highland goat; the sire’s breed is mentioned first

Means with different superscripts in each subclass within a column differ significantly (P < 0.05) from each other.

### Crossbreeding parameter estimates

#### Breed additive effect

The estimates of additive and non-additive genetic effects (heterosis and recombination) are shown in Table 3. The additive effect for LSB, LBW, LWW, LSW, and GL was estimated to be -0.004 kid, 0.08 kg, -3.18 kg, -0.54 kid, and 3.69 days, respectively. It was significant (P<0.05) for LSW, and the number of kids weaned per doe was significantly reduced due to crossing. Although it was not significant, the negative additive effect of the Boer goats in the LSB, LSW, and LWW further indicates that their effects are of maternal origin that is expressed in their crosses during the nursing period, after which they are unable to express their potential under low-input production system. This suggests that improvement in reproductive traits is not expected by crossing Boer with Central Highland goats. The failures of the Boer does to express its potential under the existing management level could explain the lower and non-significant additive contribution in this study. This situation may change in different management level, as the level of production input is a major determinant for the expression of genes and a different study, which may include more animals.

**Table 3:** Estimate of breed additive, individual heterosis, and recombination effect for doe productive traits.

Trait	CH	gB	hBCH	rBCH
LSB (kid)	1.57±0.03	-0.004±0.07^ns^	-0.03±0.04^ns^	-0.02±0.08^ns^
LBW(kg)	4.00±0.08	0.08±0.42^ns^	0.14±0.23^ns^	0.05±0.45^ns^
LWW (kid)	14.3±0.57	-3.18±4.45^ns^	-0.04±2.45^ns^	-6.20±4.72^ns^
LSW(kg)	1.40±0.03	-0.54±0.22*	0.27±0.12*	0.17±0.24^ns^
GL (days)	149.2±0.39	3.69±3.34^ns^	-2.62±1.82^ns^	-4.31±3.63^ns^

Ns, non-significant; *, P<0.05

gB, breed additive effect of Boer; hBCH, heterosis; rBCH, recombination effect

LBW, litter birth weight; LSB, litter size at birth; LWW, litter weaning weight; LSW, litter size at weaning; GL, gestation length; no., number

#### Heterosis effect

The contribution of heterosis to LSB, LWW, and GL of crossbred goats was unexpectedly negative, while the estimates for LBW and LSW were positive (Table 3). However, the mean direct heterosis was significant only for LSW, and the heterosis estimate for LSW was slightly superior to the parental breed, with 0.27 kids. The heterosis estimate obtained for LSB and LBW in this study agrees with Boujenane et al. [27] and Atashi and Izadifar [28], respectively. Likewise, the lack of a heterosis effect on the early growth traits was reported by Supakorn et al. [29], and Bondoc [30] reported that the heterocyst of some reproductive traits from 30 goat breeds was low and occasionally negative in a thorough evaluation of crossbreeding research in goats across the globe. In contrast, a significant heterosis effect for LBW and LWW was noted in the crossbreeding program with Syrian and Turkish Awassi sheep strains [31]. In addition, Fadili and Leroy [32] reported a significant heterosis effect for LSW and LWW in the crossing of D’man and Timahdite sheep breeds. The observed negative heterosis effect indicated that using Boer crossbred dams instead of pure Central Highland dams did not have any particular advantage in terms of heterosis for these traits. The expected increase in productivity due to heterosis depends on the genetic differentiation among parental breeds for a trait of interest. Heterosis increases when the allelic frequency difference between parental breeds increases [19, 28]. The absence of significant improvement in most of the reproductive traits because of heterosis in the current study could be due to low genetic differences among parents for these traits. Knowledge of the performance of parental breeds for traits of interest and the presence of significant variation among breeds is vital for a successful crossbreeding program. Thus, the choice of the parental breeds for traits of interest is quite important to maximize heterosis use and enhance the profitability of goat production. According to Thepparat et al. [33], the degree of dominance in which heterozygous exceeds both homozygotes, the amount of homozygous recessives gene, and the level of epistatic interaction between non-allelic gene pairs are the determinants of the heterosis effect. In addition, a small number of observations that may not be sufficient to disentangle the genetics from the environmental effect could be the other factor.

#### Recombination loss

The estimates of the recombination effect for LBW and LSW of crossbreds were positive, while it was negative for LSB, LWW, and GL (Table 3). The recombination effect reduced performance by -0.02 kid, -6.20 kg, and -4.31 days in LSB, LWW, and GL, respectively, although it was not significant (P>0.05) for all studied traits. This indicates that recombination losses neutralize dominance’s positive benefits on performance. This result agrees with Baas et al. [34] for pigs and Supakorn et al. [29] for early growth traits of goats. On the contrary, Haile et al. [31] noted a significant recombination effect for LBW and LWW in the crossbreeding program with Syrian and Turkish Awassi sheep strains [31]. The non-significant recombination effect in this study suggests that there should be little difference in heterosis as recombination is a loss of heterosis. Besides, the magnitude and direction of the recombination estimates suggest the possibility of synthetic breed development if there is enough advantage for crossbreeds. However, the magnitude of additive and heterosis effects in this study may not support this principle. Indeed, the negative recombination estimates for most of the examined reproductive traits in the crossbreds were not expected. Because the Boer goat has not been selected for these traits, favorable epistatic interactions between genes in different loci may not be evolved. Hence, a crossing of Boer with unimproved Central Highland goats could not significantly lose these interactions due to recombination. According to Mugambi et al. [35], if the composites were subjected to selection pressure during generations of inter se mating, retention of recombination losses would have been avoided. Thus, this could be the other reason for the non-significant influence of the recombination effect.

#### Predicted mean for different genotypes

Estimating crossbreeding parameters is essential to predict the performance of untested genotypes without actually producing the animals [21]. The expected reproductive performance as a function of genetic coefficients and crossbreeding parameters of each genotype is shown in Table 4. The LSB for indigenous goats was slightly higher than other genotypes. However, backcrosses (backcross with Boer and Central Highland) had a similar LSB. Likewise, the LSB for F1 and F2 crossbred does was found to be similar. Backcrosses with 75% and 25% Boer levels had a lower and higher LSW than other genotypes, respectively. Nevertheless, indigenous goats and F1 crossbreds had a similar LSW. Regarding LBW, the predicted means of F1s is higher than the other genotypes and lower for CH than the other genotypes. The LWW of CH was greater by 12.8, 19.7, 38.1, and 49.1% compared with B x CH, BCH x CH, B x BCH, and BCH x BCH, respectively. This indicates that LWW was decreased as a function of Boer level increases. The variation among genotypes in GL was negligible, which was less than two days.

**Table 4:** Predicted performance of doe productive traits.

Genetic group	Generation and blood level	LSB (kid)	LBW (kg)	LWW (kg)	LSW (kid)	GL (day)
CH x CH	Local	1.57	4.00	14.30	1.40	149.20
B x CH	F1 (50%)	1.54	4.18	12.67	1.40	148.43
B x BCH	BC1 (75%)	1.55	4.14	10.35	1.17	149.58
BCH x CH	BC1 (25%)	1.55	4.10	11.94	1.44	147.74
BCH x BCH	F2 (50%)	1.54	4.14	9.59	1.35	147.58

LBW, litter birth weight; LSB, litter size at birth; LWW, litter weaning weight; LSW, litter size at weaning; GL, gestation length; no., number

B, Boer goat; CH, Central Highland goat; the sire’s breed is mentioned first

### Genetic parameter estimates

#### Heritability

The variance components, heritability, and repeatability estimates for reproductive traits of Boer x Central Highland goat are presented in Table 5. Error variances were the most important source of variation for reproductive traits, implying the significant influence of systematic factors on the expression of these traits. The direct heritability estimates for examined reproductive traits varied from 0.00 in GL to 0.098 in LWB, while the permanent environmental heritability estimates ranged from 0.014 to 0.099. These estimates could be used to design breeding programs to improve reproductive traits, as the estimates for such traits are scarce, particularly in the tropics.

**Table 5:** Heritability and repeatability estimates for reproductive traits of Boer x Central Highland goats.

Estimate	σ^2^_α_	σ^2^_c_	σ^2^_e_	σ^2^_p_	h^2^	c^2^	e^2^	r
LSB	0.001	0.002	0.017	0.0197	0.050±0.04	0.099±0.15	0.851±0.10	0.149±0.09
LWB	0.058	0.010	0.522	0.590	0.098±0.07	0.018±0.05	0.884±0.07	0.116±0.07
LWW	1.993	0.330	20.96	23.28	0.086±0.08	0.014±0.06	0.900±0.08	0.099±0.07
LSW	0.002	0.008	0.116	0.128	0.018±0.03	0.068±0.08	0.914±0.07	0.086±0.06
GL	0.001	1.766	27.11	28.88	0.000±0.06	0.061±0.07	0.939±0.08	0.061±0.07

LBW, litter birth weight; LSB, litter size at birth; LWW, litter weaning weight; LSW, litter size at weaning; GL, gestation length

σ^2^_α_, additive genetic variance; σ^2^_c_, permanent environmental variance, σ^2^_e_, residual variance; σ^2^_p_, phenotypic variance; h^2^, heritability; c^2^ = σ^2^_c_ /σ^2^_p_, e^2^, error variance; r, repeatability

The heritability estimate for LSB obtained in this study is lower than the report of Mia et al. [13] for Black Bengal goats (0.08), Kebede et al. [11] for Arsi Bale goats (0.075), and Zhang et al. [9] for Boer goats (0.12). However, Mohammadi et al. [12] reported a relatively lower estimate than the current result for Raeini Cashmere goats (0.04) and Menezes et al. [14] for Boer goats (0.00). The estimated fraction of variance due to permanent environmental effects (c^2^) obtained in this study is comparable with the report of Mohammadi et al. [12] and higher than the report of Kebede et al. [11]. LSW is the other important trait in goat production, and the heritability estimate was low. The current estimate for this trait is higher than the estimate noted for Arsi Bale goat (0.005) by Kebede et al. [11] and the Markhoz goat (0.01) by Rashidi et al. [10]. On the other hand, Mia et al. [13] reported a higher estimate than the current result for the Black Bengal goat (0.13), for the Boer goat (0.10) by Zhang et al. [9], and for Egypt Nubian (0.05) by Aboul-Naga et al. [36]. A Meta-analysis [37] result noted a weighted heritability estimate of 0.05 for LSB and 0.06 for LSW of goats.

A higher LBW heritability estimate for Raeini Cashmere goats (0.16), Boer goat (0.14), and Arsi Bale goat (0.126) than the current result were noted by Mohammadi et al. [12], Zhang et al. [9] and Kebede et al. [11], respectively. However, the estimates in this study are comparable with the report of Mia et al. [13] for Black Bengal goats and higher than the estimate reported by Rashidi et al. [10] for Markhoz goats. The heritability estimate for LWW in this study is higher than the previous findings of Rashidi et al. [10], Kebede et al. [11], and Jembere et al. [37] but lower than the result noted for pure Boer goats [14]. The heritability estimate for GL was lower than that for other goat breeds [9, 13]. A variation of estimates among studies is likely due to the difference in analytical models, data structure, genetic composition in the population [9], and systematic factors examined. Heritability estimates for a trait of interest indicate the magnitude of expected genetic progress through selection. Despite their economic importance, the improvement of reproductive traits through selection would be small, as heritability and additive genetic variance estimates indicate.

#### Repeatability

The repeatability estimates for LSB, LWB, LWW, LSW, and GL were 0.149, 0.116, 0.099, 0.086, and 0.061, respectively (Table 5), which is found within a low range (<0.20). The repeatability estimate for LSB obtained in this study agrees with the findings of different scholars [11, 12, 36] for different breeds and is higher than the estimate found for pure Boer goats [14]. Higher repeatability estimates for LSW than the current estimate were reported for different breeds [9, 11, 12], and a lower estimate (0.09) was noted for the Egypt Nubian goat breed [36]. Menezes et al. [14] reported relatively high repeatability estimates for LBW and LWW for the Boer goat, Kebede et al. [11] for the Arsi Bale goat, Mohammadi et al. [12] for the Raeini Cashmere goat and Zhang et al. [9] for Boer goat. The difference in sample size, breed, management conditions, and the number of random and systematic factors considered in the estimation procedures could explain the variation of repeatability estimates among different studies. If the animal has a higher repeatability value for a certain characteristic, it can be decided whether to keep or cull the animals based on the first record of observation; if the has a lower repeatability value, more than one observation on the same character is required. Therefore, the finding of this study indicates that multiple records have to be used to decide whether to cull or select breeding females.

## Conclusions

The effect of breed additive, heterosis, and recombination was non-significant for most of the investigated reproductive traits. This result suggests that Boer x Central Highland dams have no considerable advantage over Central Highland dams regarding reproductive performance under the same environmental conditions. The low heritability estimates suggest that selection based on own performance may result in slow genetic progress, and the repeatability estimates indicate that multiple records have to be used to decide whether to cull or select. Future studies in populations with better data size and structure may further investigate the impact of crossing with Boer goats on reproductive traits in terms of additive genetic and non-additive effects.

## Supporting information

S1 Data Reproductive data. (XLSX)

## References

[1] Schiermieste LN. Estimation of Breed Specific Heterosis Effects for Birth, Weaning and Yearling Weight in Cattle. University of Nebraska, 2014; p. 94. M.Sc. Thesis. http://digitalcommons.unl.edu/animalscidiss/94. 10.2527/jas.2014-8493 25568356

[2] Williams JL, Aguilar I, Rekaya R, Bertrand JK. Estimation of breed and heterosis effects for growth and carcass traits in cattle using published crossbreeding studies. J. Anim. Sci. 2014; 88: 460–466. 10.2527/jas.2008-1628. 19820043

[3] Tesema Z, Kebede D. Do Crossbreeding using Exotic Breeds in Goat is the Right Solution for a Low-input Production System in Ethiopia?: A Review. Agricultural Reviews. 2022; 43(1): 11–19. doi: 10.18805/ag.R-183

[4] Tesema Z, Alemayehu K, Getachew T, Kebede D, Deribe B, Taye M, et al. Estimation of genetic parameters for growth traits and Kleiber ratios in Boer x Central Highland goat. Trop. Anim. Health Prod. 2020a; 52: 3195–3205. doi: 10.1007/s11250-020-02345-z 32748084

[5] Tesema Z, Alemayehu K, Kebede D, Getachew T, Kefale A, Deribe B. Reproductive performance and milk production of Central Highland and Boer x Central Highland goats. Heliyon. 2020b; 6 (2020): e05836. doi: 10.1016/j.heliyon.2020.e05836 33409396 PMC7773859

[6] Tesema Z, Alemayehu K, Kebede D, Getachew T, Deribe B, Tilahun M, et al. Evaluation of growth and efficiency-related traits of different levels of Boer x Central Highland crossbred goats. Heliyon. 2021; 7 (2021): e08184. doi: 10.1016/j.heliyon.2021.e08184 34765763 PMC8569402

[7] Gebrelul S, Satin LS, Iheanacho M. Genetic and non-genetic effects on growth and mortality of Alpine, Nubian and crossbred kids. Small Ruminant Res. 1994; 13: 169–76. 10.1016/0921-4488(94)90093-0.

[8] Haile A, Joshi BK, Ayalew W, Tegegne A, Singh A. Genetic evaluation of Ethiopian Boran cattle and their crosses with Holstein Friesian for growth performance in central Ethiopia. J. Anim. Breed. Genet. 2011; 128 (2011): 133–140. doi: 10.1111/j.1439-0388.2010.00882.x 21385228

[9] Zhang CY, Chen SL, Li X, Xu DQ, Zhang Y, Yang LG. Genetic and phenotypic parameter estimates for reproduction traits in the Boer dam. Livest. Sci. 2009; 125: 60–65. 10.1016/j.livsci.2009.03.002.

[10] Rashidi A, Bishop SC, Matika O. Genetic parameter estimates for pre weaning performance and reproduction traits in Markhoz goats. Small Rumin. Res. 2011; 100:100–106. 10.1016/j.smallrumres.2011.05.013.

[11] Kebede K, Haile A, Dadi H, Alemu T. Genetic and phenotypic parameter estimates for reproduction traits in indigenous Arsi-Bale goats. Trop Anim Health Prod. 2012; 44: 1007–1015. doi: 10.1007/s11250-011-0034-8 22160562

[12] Mohammadi H, Shahrebabak MM, Shahrebabak HM. Genetic parameter estimates for growth traits and prolificacy in Raeini Cashmere goats. Trop Anim Health Prod. 2012; 44: 1213–1220. doi: 10.1007/s11250-011-0059-z 22213036

[13] Mia MM, Khandoker MAMY, Husain SS, Faruque MO, Notter DR. Estimation of genotypic and phenotypic parameters for some reproductive traits of Black Bengal does. Iranian journal of applied animal science. 2013; 3(4): 829–83.

[14] Menezes LM, Sousa WH, Cavalcanti-Filho EP, Gama LT. Genetic parameters for reproduction and growth traits in Boer goats in Brazil. Small Ruminant Research. 2016; 136: 247–256. 10.1016/j.smallrumres.2016.02.003.

[15] Belay S, Gebru G, Godifey G, Brhane M, Zenebe M, Hagos H, et al. Reproductive performance of Abergelle goats and growth rate of their crosses with Boer goats. Livestock Research for Rural Development. 2014; 26(1). http://www.lrrd.org/lrrd26/1/bela26005.htm.

[16] Deribe B, Tilahun M, Lakew M, Belayneh N, Zegeye A, Walle M, et al. On-station growth performance of crossbred goats (Boer x Central Highland) at Sirinka, Ethiopia. Asian Journal of Animal Sciences, 2015; ISSN 1819-1878 / doi: 10.3923/ajas.2015

[17] SAS. SAS user’s guide version 9.1: Statistics. Cary, NC: SAS Institute Inc. 2002.

[18] Robison OW, McDaniel BT, Ricon EJ. Estimation of direct and maternal additive and heterosis effects from crossbreeding experiments in animals. J. of Anim. Sci. 1982; 52: 44–50. 10.2527/jas1981.52144x. 7240038

[19] Dickerson GE. Inbreeding and heterosis in animals. Proceedings Animal Breeding and Genetic Symposium. In Honour of Dr. J.L. Lush, American Society of Animal Science and American Dairy Science, Blackbourg, VA, 1973; pp. 54–77.

[20] Wolf J, Distl O, Hyamek J, Grosshans T, Seeland G. Crossbreeding in farm animals. V. Analysis of crossbreeding plans with secondary crossbred generations. J. Anim. Breed. Genet. 1995; 112: 81–94. 10.1111/j.14390388.1995.tb00545.x.

[21] Demeke S, Neser FWC, Schoeman SJ. Early growth performance of *Bos taurus* x *Bos indicus* cattle crosses in Ethiopia: estimation of individual crossbreeding effects. J. Anim. Breed. Genet. 2003; 120 (2003): 245–257. 10.1046/j.14390388.2003.00392.x.

[22] Lynch M, Walsh B. Genetics and Analysis of Quantitative Traits. Sinauer Associates, Inc., Sunderland, MA, USA; 1998.

[23] Meyer K. WOMBAT—a tool for mixed model analyses in quantitative genetics by restricted maximum likelihood (REML). J. Zhejiang Univ. 2007; 8(11): 815–821. doi: 10.1631/jzus.2007.B0815 17973343 PMC2064953

[24] Nguluma A, Leite-Browning ML, Browning R Jr. Comparison of Boer-Cross and foundation breeds for meat goat doe fitness in the humid subtropics. Livest. Res. Rural Dev. 2013; 25(3). http://www.lrrd.org/lrrd25/3/ngul25038.htm.

[25] Khanal P. Influence of Crossbreeding and Non Genetic Factors on Doe Fitness Traits of Boer F1 and Foundation Breeds in southeastern United States. MSc. Thesis. Tennessee State University, United State, 2016; pp. 68.

[26] Mustefa A, Banerjee S, Gizaw S, Taye M, Getachew T, Areaya A, et al. Reproduction and survival analysis of Boer and their crosses with Central Highland goats in Ethiopia. Livest. Res. Rural Dev. 2019; 31(10). http://www.lrrd.org/lrrd31/10/amine31166.html.

[27] Boujenane L, Bradford GE, Famula TR. Inheritance of liter size and its components in crosses between the D’man and Sardi breeds of sheep. J. Anim. Sci. 1991; 69: 517–524. 10.2527/1991.692517x. 2016181

[28] Atashi H, Izadifar J. Estimation of individual heterosis for lamb growth in Ghezal and Mehraban sheep. Iranian Journal of Applied Animal Science. (2012); 2(2): 127–130.

[29] Supakorn C, Pralomkarn W, Tumwasorn S. Estimation of Additive, Non Additive Gene Effects and Genetic Parameters on Pre-Weaning Growth Traits in Meat Goats in Southern Thailand. Walailak J Sci & Tech. 2011; 8(1): 41‐50. https://wjst.wu.ac.th/index.php/wjst/article/view/10.

[30] Bondoc OL. The Philippine goat breed registry in relation to genetic improvement and conservation. The Phil Agric Sci. 2005;88:179–191.

[31] Haile A, Hilali M, Hassen H, Lobo RNB, Rischkowsky B. Estimates of genetic parameters and genetic trends for growth, reproduction, milk production and milk composition traits of Awassi sheep. Animal. 2019; 13(2): 240–247. doi: 10.1017/S1751731118001374 29954467

[32] Fadili M E, Leroy PL. Estimation of additive and non-additive genetic parameters for reproduction, growth and survival traits in crosses between Moroccan D’man and Timahdite sheep breeds. J. Anim. Br. Genet. 2001; 341–353. 10.1046/j.1439-0388.2001.00297.x.

[33] Thepparat M, Duangjinda M, Tumwasorn S. Random heterosis effects on genetic parameters, estimation of birth weight, and Kleiber ratio in a population admixture of Thailand goats. Livest Sci. 2012; 147: 27–32. 10.1016/j.livsci.2012.03.015.

[34] Baas TJ, Christian LL, Rothschild MF. Heterosis and Recombination Effects in Hampshire and Landrace Swine: I. Maternal Traits. J. Anim. Sci. 1992; 70: 89–98. doi: 10.2527/1992.70189x 1582925

[35] Mugambi JN, Wakhungu JW, Inyangala BO, Muhuyi WB, Muasya T. Evaluation of the performance of the Kenya Dual Purpose Goat composites: Additive and non-additive genetic parameters. Small Ruminant Research. 2007; 72 (2007): 149–156. 10.1016/j.smallrumres.2006.10.001.

[36] Aboul-Naga AM, Hamed A, Shaat I, Mabrouk MMS. Genetic improvement of Egyptian Nubian goats as sub–tropical dairy prolific breed. Small Ruminant Research. 2012;102:125–130. 10.1016/j.smallrumres.2011.06.014.

[37] Jembere T, Dessie T, Rischkowsky B, Kebede, K, Okeyo AM, Haile A. Meta-analysis of average estimates of genetic parameters for growth, reproduction and milk production traits in goats. Small Ruminant Research. 2017; 153 (2017): 71–80. 10.1016/j.smallrumres.2017.04.024.

