# Correction: Estimation of crossbreeding and genetic parameters for reproductive traits of Boer x Central Highland goats in Ethiopia

**DOI:** 10.1371/journal.pone.0318864

**Published:** 2025-01-30

**Authors:** Zeleke Tesema, Belay Deribe, Mekonnen Tilahun, Alemu Kefale, Getachew Worku Alebachew, Kefyalew Alemayehu, Tesfaye Getachew, Damitie Kebede, Mengistie Taye, Solomon Gizaw

In the Statistical analysis subsection of Materials and Methods, there is an error in the fifth sentence of seventh paragraph. The correct sentence is: Repeatability (r) = σ^2^_*a*_ + σ^2^_c_ / σ^2^_p_, where σ^2^_*a*_ is the direct additive genetic variance, σ^2^_c_ is the permanent environmental variance and σ^2^_p_ is phenotypic variance.
